# Multimodal Data Sharing, Analysis Workflows, and Biosample Discovery in a Global Consortium Approach: How the Global Research and Imaging Platform Can be Leveraged to Enhance Research and Collaboration at ADRCs and Dementia Centers Worldwide

**DOI:** 10.1002/alz70856_098642

**Published:** 2025-12-24

**Authors:** Farhad Imam

**Affiliations:** ^1^ Gates Ventures, Seattle, WA, USA

## Abstract

**Background:**

Lack of end‐to‐end, multimodal collaborative data analysis platforms are a key gap to fill for the dementia research community. The vast expanse of neurodegenerative disease data available today offers an unprecedented opportunity for deep analysis to provide the types of groundbreaking insights that could significantly impact Alzheimer's and dementia‐related outcomes. The Global Research and Imaging Platform (GRIP) is addressing these challenges via its **open access, user‐friendly secure cloud environment** (www.grip‐research.org).

**Method:**

GRIP aims to improve productivity by freeing time for researchers to focus on their most important work, optimize spending via open access (no licensing fees), and facilitate far‐reaching collaborations and larger‐scale analyses that lead to faster results. GRIP's five key primary product components – discovery, access requests, data sharing, workspaces, and workflows – are all supported by a modular, underlying infrastructure built to support biomedical imaging research. To date, GRIP has partnered with **11 leading ADRC institutions** and additional partners across the U.S. and Europe to develop a set of **17 modular tools** that are incorporated into coherent workflows across **imaging, digital, and ‘omics data**. GRIP's partner institutions are organized into six distinct working groups that meet on a regular cadence to actively collaborate across multiple research centers to build cross‐compatible workflows and modules. Through co‐creating community data analysis tools with best‐in‐class partners, we are optimizing GRIP for key researcher needs.

**Result:**

**GRIP's dementia‐focused platform offering, scheduled for launch at the start of Q2 2025,** is providing over a dozen analysis workflows for neuroimaging, digital voice, and ‘omics data in a streamlined, secure cloud environment for individual researchers and large, international consortia alike.

**Conclusion:**

GRIP is intended to save money and time for both researchers and funders. More importantly, it is intended to facilitate cutting‐edge research that isn’t happening now because the infrastructure for it doesn’t exist. That research could lead to insights that result in better options for the 50 million people worldwide living with Alzheimer's and related dementias.

